# Juvenile Nasopharyngeal Angiofibroma in an Adult Patient: A Rare Presentation with Fahr Syndrome and Multiple Comorbidities—A Case Report and Literature Review

**DOI:** 10.3390/diagnostics16091327

**Published:** 2026-04-28

**Authors:** Sigita Zālīte, Karīna Čudare, Kalvis Vērzemnieks, Sergejs Pavlovičs, Kārlis Kupčs, Ingus Vilks, Tatjana Tone, Inese Briede, Arturs Balodis

**Affiliations:** 1 Faculty of Medicine, Riga Stradins University, LV-1007 Riga, Latvia; sigita.sk14@gmail.com; 2 Faculty of Medicine and Life Sciences, University of Latvia, LV-1004 Riga, Latvia; cudare.karina@gmail.com (K.Č.); kalvisverzemnieks@gmail.com (K.V.); 3 Institute of Diagnostic Radiology, Pauls Stradins Clinical University Hospital, LV-1002 Riga, Latviakkupcs@gmail.com (K.K.); 4 Department of Radiology, Riga Stradins University, LV-1007 Riga, Latvia; 5 Clinic of Otolaryngology, Pauls Stradins Clinical University Hospital, LV-1002 Riga, Latvia; ingus.vilks@rsu.lv; 6 Department of Otorhinolaryngology, Riga Stradins University, LV-1002 Riga, Latvia; 7 Department of Pathology, Pauls Stradins Clinical University Hospital, LV-1002 Riga, Latvia; tatjana.tone@stradini.lv; 8 Department of Pathology, Riga Stradins University, LV-1007 Riga, Latvia; 9 Laboratory Service, Department of Pathology, Riga East University Hospital, LV-1007 Riga, Latvia

**Keywords:** nasopharyngeal angiofibroma, hypoparathyroidism, Fahr-like intracranial calcifications, endoscopic resection

## Abstract

**Background and Clinical Significance:** Juvenile nasopharyngeal angiofibroma (JNA) is a benign but locally aggressive vascular tumor, classically affecting adolescent males. Diagnosis in adulthood is exceptionally uncommon and may mimic other vascular or malignant nasopharyngeal lesions. This patient also had chronic hypocalcemia with Fahr-like intracranial calcifications secondary to long-standing postoperative hypoparathyroidism after thyroid carcinoma treatment. To our knowledge, this coexistence has not been previously reported. **Case Presentation:** A 34-year-old Caucasian male with papillary thyroid carcinoma treated with total thyroidectomy developed postoperative hypoparathyroidism with chronic hypocalcemia and Fahr-like intracranial calcifications. During admission for acute respiratory insufficiency due to tracheostomy dysfunction, imaging revealed a 37 × 33 × 32 mm heterogeneous, hypervascular nasopharyngeal mass extending into the right pterygopalatine fossa (PPF) with bone remodeling and focal bony dehiscence. Digital subtraction angiography demonstrated a markedly hypervascular tumor, predominantly supplied by branches of the right internal maxillary artery (via the sphenopalatine artery). Endoscopic resection was performed, and histopathology confirmed JNA. Most JNA cases occur between 7 and 19 years of age; presentations in men older than 30 years are rare and often generate diagnostic uncertainty, particularly when differentiating from nasopharyngeal carcinoma or other lesions. In adults, magnetic resonance imaging/computed tomography for assessment of local extent and angiography for vascular mapping are key to minimizing hemorrhagic risk. The concurrent endocrine disorder emphasizes the need for multidisciplinary perioperative metabolic optimization, without implying a pathophysiological link. **Conclusions:** This report illustrates JNA diagnosed in adulthood in a male with Fahr-like intracranial calcifications secondary to chronic hypoparathyroidism. It highlights the necessity of considering JNA in the differential diagnosis of hypervascular nasopharyngeal masses in adults, especially in patients with complex comorbidities.

## 1. Introduction

Juvenile nasopharyngeal angiofibroma (JNA) is a rare, benign yet locally aggressive vascular tumor of the nasopharynx, accounting for less than 0.5% of all head and neck neoplasms [1,2,3]. It typically arises in adolescent males between 7 and 19 years of age and manifests with nasal obstruction, recurrent epistaxis, and in advanced cases, craniofacial deformity or skull base involvement [3,4]. Diagnosis of JNA in adulthood is uncommon and often raises diagnostic uncertainty, as the clinical and radiological presentation may overlap with other vascular or malignant tumors of the sinonasal region [5].

Intracranial calcifications with a bilateral basal ganglia–predominant distribution may reflect primary familial brain calcification (PFBC) or secondary (“Fahr-like”) calcifications related to identifiable etiologies, including metabolic and endocrine disorders [6]. The term “Fahr disease” is commonly used in the literature to describe PFBC, whereas “Fahr syndrome” refers to secondary basal ganglia calcifications in the setting of an underlying cause (most often disturbances of calcium–phosphate metabolism, such as hypoparathyroidism).

In this report, we describe an adult patient with postsurgical hypoparathyroidism complicated by Fahr-like intracranial calcifications who was subsequently found to have a vascular nasopharyngeal mass diagnosed as JNA. This case is presented to highlight diagnostic reasoning and perioperative management considerations in a patient with complex comorbidity and relevant imaging findings, without implying a direct pathophysiological association between these entities.

In the present case, the patient had previously undergone total thyroidectomy for papillary thyroid carcinoma, which led to postoperative hypoparathyroidism and chronic hypocalcemia. As a consequence of prolonged calcium-phosphate imbalance, the patient developed Fahr-like intracranial calcifications, characterized by bilateral deposits predominantly involving the basal ganglia, thalamus, and cerebellum. While both juvenile nasopharyngeal angiofibroma and Fahr syndrome-like intracranial calcifications have been described independently [2,5,7], their coexistence in a single patient has not, to our knowledge, been previously reported. Accordingly, we report JNA diagnosed in adulthood in a male with postsurgical hypoparathyroidism and Fahr-like intracranial calcifications, emphasizing the need to maintain JNA in the differential diagnosis of hypervascular nasopharyngeal masses in adults, while recognizing that current evidence does not support a causal link between these conditions.

## 2. Case Presentation

**Figure 1:**
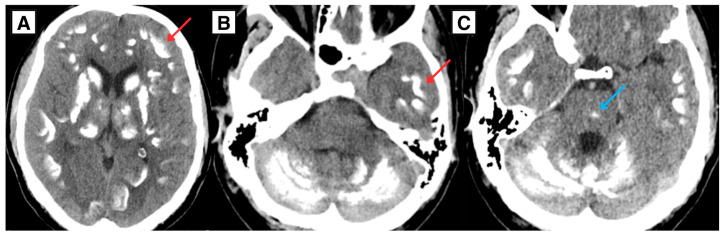
Non-contrast axial brain CT demonstrates extensive intracranial calcifications with a basal ganglia predominant distribution, consistent with Fahr-like intracranial calcifications. (**A**) Symmetric calcifications in the basal ganglia and thalami. (**B**) Extensive calcifications in the subcortical white matter and cerebellar structures. (**C**) Calcification involving the midbrain. Red arrow: symmetric subcortical white matter calcifications. Blue arrow: midbrain calcification.

**Figure 2:**
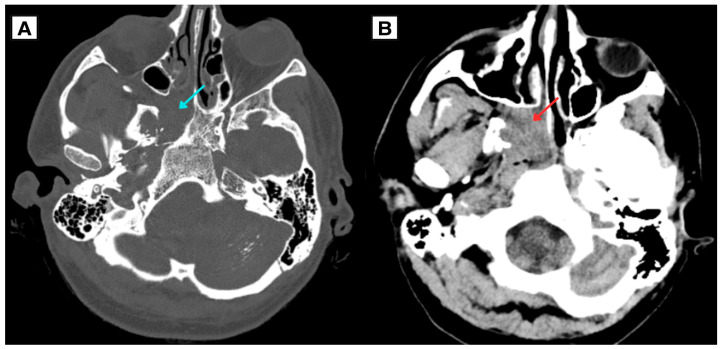
CT of the nasopharyngeal region. (**A**) Axial bone window demonstrates right-sided dilatation of the pterygoid (vidian) canal (blue arrow) and remodeling of the anterior wall of the sphenoid sinus. (**B**) Incidental suspicion of a right-sided nasopharyngeal mass (red arrow) with extension toward the pterygopalatine fossa.

A 34-year-old Caucasian male with a complex endocrine and oncological history was referred to a tertiary university hospital in January 2025 due to acute respiratory insufficiency secondary to tracheostomy dysfunction. Initial laboratory testing showed severe hypocalcemia (serum calcium 1.18 mmol/L). During the initial work-up, brain computed tomography (CT) demonstrated extensive intracranial calcifications involving the basal ganglia, thalami, subcortical white matter, cerebellum (including the dentate nuclei), and brainstem—findings consistent with Fahr-like intracranial calcifications in the setting of chronic postsurgical hypoparathyroidism with longstanding calcium–phosphate imbalance (Figure 1) [3,7]. The same CT incidentally suggested a right-sided nasopharyngeal mass (Figure 2). The right pterygoid canal was dilated compared with the contralateral side, an imaging feature suggestive of tumor extension toward the vidian canal region and involvement of the pterygopalatine fossa, as described in JNA. No obvious mass was clinically visible on routine otorhinolaryngological examination of the nose and nasopharynx, and the lesion was first suspected on imaging.

**Figure 3:**
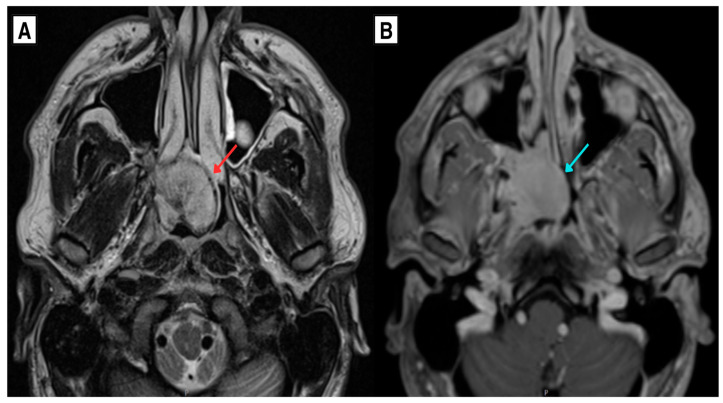
MRI of the nasopharyngeal region. (**A**) Axial T2-weighted image demonstrates a heterogeneous mass centered in the right nasopharynx (red arrow) with internal hypointense foci and extension into the right pterygopalatine fossa (blue arrow), associated with bony remodeling. (**B**) Axial post-contrast T1-weighted image demonstrates avid enhancement, consistent with a hypervascular lesion.

**Figure 4:**
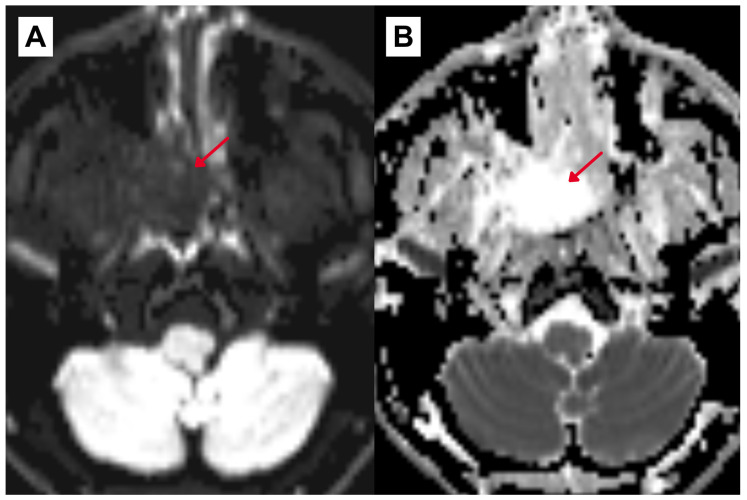
Diffusion-weighted imaging (DWI) and apparent diffusion coefficient (ADC) map of the right nasopharyngeal mass (red arrow). (**A**) Axial DWI shows no diffusion restriction. (**B**) Axial ADC map confirms the absence of low ADC values.

Subsequent contrast-enhanced magnetic resonance imaging (MRI) in February 2025 revealed a 37 × 33 × 32 mm heterogeneous lesion centered in the right nasopharynx with extension into the right pterygopalatine fossa, associated with bone remodeling and focal osseous erosion/dehiscence of the medial wall of the maxillary sinus and anterior wall of the sphenoid sinus (Figure 3 and Figure 4). The lesion appeared vascular in post-contrast T1-weighted imaging and demonstrated flow–voids on T2-weighted sequences, without diffusion restriction on diffusion-weighted imaging (DWI). Susceptibility-weighted imaging (SWI) showed the presence of blood products. No signs of hydrocephalus or significant parenchymal volume loss were observed.

The patient’s medical history included papillary thyroid carcinoma diagnosed at age 8 with pulmonary metastases (T4N1bM1), treated in 1998 with total thyroidectomy followed by radioactive iodine therapy and chemotherapy. Serial chest CT examinations performed over the last 4 years demonstrated multiple pulmonary nodules without significant interval change or radiological evidence of progression. The latest available laboratory assessment showed low thyroglobulin levels, which, together with the stable imaging findings, did not suggest active recurrence during the reported period.

Postoperatively, he developed bilateral recurrent laryngeal nerve palsy requiring tracheostomy, chronic postsurgical hypoparathyroidism with recurrent hypocalcemia, and hypothyroidism treated with levothyroxine. The patient had inconsistent adherence to active vitamin D therapy. The last documented calcitriol prescription dated from 2021 despite recurrent hypocalcemic episodes requiring hospitalization. In 2009, he was hospitalized following a seizure due to severe hypocalcemia, and long-term calcium and vitamin D supplementation was emphasized. Thereafter, hypocalcemic episodes recurred intermittently, particularly during periods of poor oral intake. Apart from the documented neurological and metabolic manifestations, no other systemic complications of chronic hypoparathyroidism were identified in the available records, including nephrocalcinosis, nephrolithiasis, cataracts, or skeletal calcifications. As the patient had no symptoms related to other organ systems, whole-body CT was not performed to avoid unnecessary radiation exposure.

**Figure 5:**
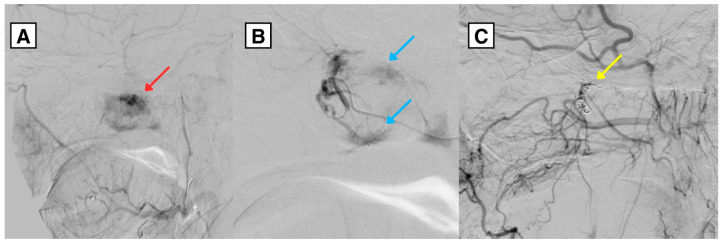
Digital subtraction angiography of the external carotid circulation. (**A**) Right external carotid artery angiogram demonstrates a markedly hypervascular nasopharyngeal mass (red arrow), predominantly supplied by branches of the right internal maxillary artery via the sphenopalatine artery. (**B**) Selective microcatheterization of the sphenopalatine artery (blue arrow) followed by embolization. (**C**) Contralateral external carotid artery angiogram demonstrates no residual tumor blush/hypervascularity in the region of the lesion; the embolization coil is indicated by the yellow arrow.

Digital subtraction angiography (DSA) performed on 20 February 2025 confirmed that the lesion was hypervascular and supplied by branches of the right internal maxillary artery via the sphenopalatine artery (Figure 5), consistent with JNA. Under general anesthesia, a 6F introducer sheath was placed via the right common femoral artery. The right external carotid artery was catheterized using a 6F guiding catheter. Selective microcatheterization of the feeding branch was performed followed by embolization with 355–500 μm polyvinyl alcohol (PVA) particles, achieving substantial devascularization. The feeding sphenopalatine artery was additionally embolized using a 6 × 20 mm coil. Control DSA from the left external carotid artery demonstrated no residual hypervascularity in the region of the lesion.

During hospitalization, endocrine therapy was optimized with calcitriol 0.5 μg twice daily and calcium carbonate 3000 mg/day, titrated to serum calcium. Levothyroxine was increased from 75 μg to 100 μg daily due to subclinical hypothyroidism. The patient remained clinically stable and neurologically intact.

**Figure 6:**
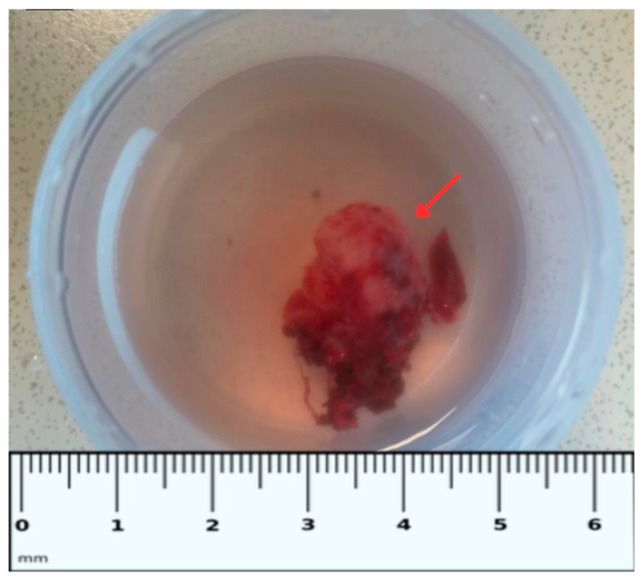
Macroscopic appearance of the resected tumor specimen (red arrow) following endoscopic resection, measuring 16 × 24 mm (anteroposterior × laterolateral).

**Figure 7:**
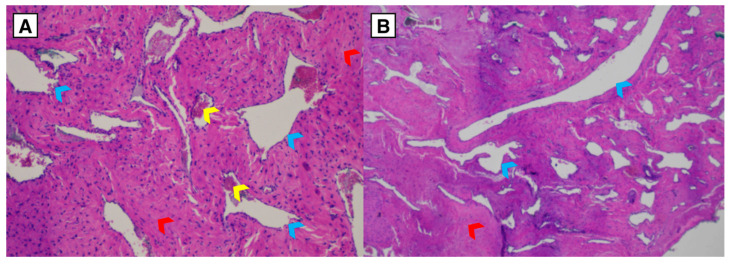
Hematoxylin and eosin stain of juvenile nasopharyngeal angiofibroma. The lesion is composed of irregular, variably sized vascular channels (blue arrowhead) with variable wall thickness, embedded in a collagenous/fibrous stroma (red arrowhead). Fibrinous thrombi (yellow arrowhead) are present within a subset of vessels. (**A**) Low-power view (original magnification 10×). (**B**) Low-power view (original magnification 10×).

Preoperative management included nasal cavity debridement and endoscopic evaluation. Endoscopic surgical resection of the nasopharyngeal mass was performed in May 2025 under neuronavigation guidance using a functional endoscopic sinus surgery (FESS) approach. The gross specimen is shown in Figure 6. Histopathology confirmed JNA (Figure 7).

**Figure 8:**
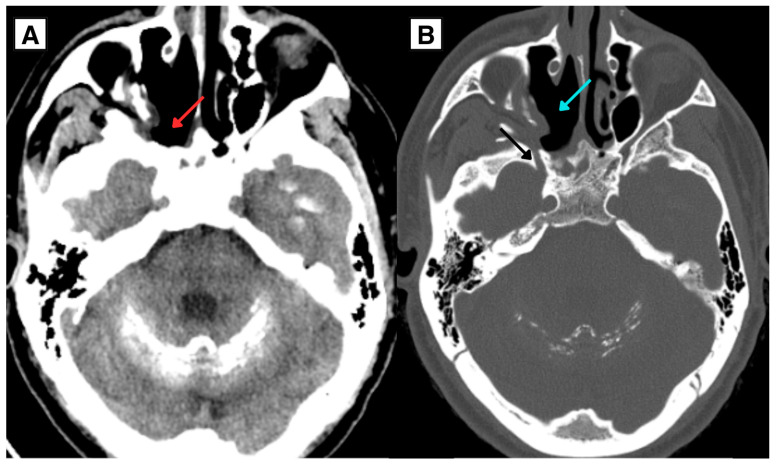
Follow-up non-contrast CT of the head/paranasal sinuses 3 months after endoscopic resection of the right-sided nasopharyngeal mass. (**A**) Axial CT demonstrates postoperative changes without evidence of residual or recurrent mass (red arrow). (**B**) Axial CT demonstrates postoperative bony defects in the medial wall of the maxillary sinus (blue arrow) and enlarged foramen rotundum (black arrow). Extensive intracranial calcifications persist.

At follow-up in June 2025, the patient was clinically stable with well-healed surgical sites, no evidence of recurrence on imaging, and improved metabolic control under the revised endocrine regimen. Follow-up imaging on 11 August 2025 (3 months post-resection) demonstrated postoperative defects in the medial wall of the maxillary sinus and the ventral-inferior wall of the right sphenoid sinus chamber (Figure 8) without evidence of pathological lymphadenopathy. Extensive intracranial calcifications persisted as previously described.

From an endocrine perspective, the patient’s long-term prognosis depends mainly on adherence to lifelong calcium and active vitamin D supplementation together with regular biochemical monitoring. The principal ongoing clinical risk remains recurrent symptomatic hypocalcemia, particularly during periods of poor oral intake or poor treatment adherence. At the latest available follow-up, no additional functional limitations attributable to hypoparathyroidism were documented beyond the need for continued therapy and monitoring.

## 3. Discussion

JNA typically manifests during adolescence, with most cases diagnosed between the ages of 7 and 19 [1,2,4]. This demographic pattern has been reaffirmed in recent reviews, emphasizing the hormonal and vascular dependency of the tumor on androgenic stimulation [3,8,9]. Nevertheless, rare cases diagnosed in adulthood have been reported, with presentations in men older than 30 years and occasional deviations from the classic adolescent clinical profile [10,11,12]. These adult cases, similar to the present one, frequently prompt diagnostic uncertainty because radiologic and clinical features can overlap with nasopharyngeal carcinoma and other skull-base lesions, including meningioma [10,13]. To contextualize diagnosis in adulthood and highlight shared diagnostic and management patterns, we summarize reported cases of JNA in patients ≥30 years [14,15,16,17,18,19,20,21,22,23,24,25,26] in Table 1.

Although the term “juvenile” reflects the typical age group, published adult cases confirm that nasopharyngeal angiofibroma can rarely occur beyond the third decade. Recent reports also emphasize delayed recognition and diagnostic anchoring toward malignancy, given that adult patients fall outside the expected demographic [22,24].

From an imaging standpoint, MRI and CT are crucial in distinguishing JNA from other nasopharyngeal lesions [5,13,27]. Characteristic MRI features include avid enhancement and multiple flow-voids, supporting a confident preoperative diagnosis and, importantly, helping avoid biopsy given the hemorrhagic risk. Standardized staging and careful mapping of extensions (pterygopalatine/infratemporal fossa and skull base involvement) further inform surgical strategy and bleeding-risk anticipation [28,29]. In our patient, intense enhancement, bone remodeling, and prominent flow-voids supported a vascular tumor, while digital subtraction angiography confirmed predominant supply from branches of the right internal maxillary artery via the sphenopalatine artery [5,13]. Recent imaging-based case reports describe comparable patterns of flow-voids and bone remodeling, reinforcing the role of MRI in differentiating JNA from invasive malignancies [13].

Management of JNA in adult patients generally follows the same principles as in adolescents with endoscopic resection being the preferred approach for localized disease [1,5,10,11]. Recent studies support endoscopic resections for improved visualization and minimal morbidity, as also employed in this patient’s treatment [1,5,11]. However, given the patient’s multiple comorbidities, perioperative management required close coordination among radiology, otolaryngology, endocrinology, and anesthesiology teams, particularly to stabilize calcium homeostasis and thyroid hormone replacement.

In this case, the patient had a long-standing history of papillary thyroid carcinoma treated with total thyroidectomy and radioactive iodine therapy, complicated by postoperative hypoparathyroidism. This resulted in chronic hypocalcemia and widespread intracranial calcifications consistent with Fahr–like changes secondary to hypoparathyroidism. Fahr syndrome secondary to chronic hypoparathyroidism has been well documented [6,7], though its coexistence with a nasopharyngeal vascular neoplasm has not been previously reported. Persistent hypocalcemia despite supplementation underscores the long-term consequences of endocrine dysfunction following thyroid surgery and highlights the need for careful metabolic optimization in complex surgical patients.

Chronic postsurgical hypoparathyroidism is a well-recognized long-term complication after total thyroidectomy. Contemporary guidance emphasizes maintaining serum calcium within a target range that prevents symptoms while minimizing hypercalciuria and renal complications, supported by structured biochemical monitoring and perioperative adjustment of calcium and active vitamin D therapy [30,31,32]. Fahr–like intracranial calcifications are repeatedly linked to disorders of calcium–phosphate metabolism—most commonly hypoparathyroidism—and published reviews and case series highlight that neurological manifestations can be heterogeneous and may partially improve with metabolic correction [33,34]. In complex surgical candidates, these data support proactive endocrine optimization as a safety-critical component of perioperative planning.

In our patient, JNA diagnosed in adulthood coexisted with chronic postsurgical hypoparathyroidism, persistent hypocalcemia, and Fahr-like intracranial calcifications, a combination of clinically distinct conditions not previously described in the literature [6,7]. While adolescent JNA is often discussed in relation to hormonal influences [3,8,9], the coexistence of JNA with hypoparathyroidism-related Fahr-like calcifications should be interpreted as comorbidity rather than evidence of a shared pathophysiological mechanism. In the absence of prior sinonasal imaging, tumor onset cannot be determined; therefore, this case is best described as JNA diagnosed in adulthood rather than proven adult-onset disease. The highly vascular nature of JNA demands careful preoperative imaging and planning, often including preoperative embolization to reduce intraoperative bleeding risk [1,5,27].

Overall, the multidisciplinary strategy in this case illustrates how integrated metabolic control and precise image-guided surgery can facilitate favorable outcomes in complex endocrine-oncologic patients. In the context of the available literature, the present case adds to current knowledge in two ways: first, it reinforces that JNA remains a relevant diagnostic consideration in adult males with hypervascular nasopharyngeal masses, where misclassification as malignancy is a recognized risk; and second, it documents an unusual comorbidity intersection—JNA diagnosed in adulthood in a patient with chronic postsurgical hypoparathyroidism and Fahr-like intracranial calcifications—an association not previously highlighted in published adult JNA reports and metabolic calcification reviews. Rather than implying causality, this case highlights the importance of endocrine and metabolic optimization in patients with relevant comorbidities undergoing treatment for atypical hypervascular head-and-neck tumors.

## 4. Conclusions

Overall, this case illustrates three key points: (1) the rare diagnosis of juvenile nasopharyngeal angiofibroma in adulthood in a patient with long-standing endocrine and metabolic disease; (2) the previously unreported comorbidity of hypoparathyroidism-related Fahr-like intracranial calcifications in a patient with JNA; and (3) a favorable outcome achieved through multidisciplinary collaboration and advanced image-guided intervention. These findings emphasize the need for systematic metabolic assessment and optimization in adults with atypical hypervascular nasopharyngeal lesions and highlight the necessity of a tailored perioperative strategy integrating surgical, radiological, and endocrine management.

## Figures and Tables

**Table 1:** Nasopharyngeal angiofibroma diagnosed in adulthood (≥30 years) reported in the literature and the present case.

Author (Year)	Age/Sex	Presentation	Imaging Findings	Surgery	Outcome
Shah et al. [14] (2000)	32/M	Right nasal mass with obstruction (2 mo) and intermittent epistaxis; progressive protrusion	X-ray: homogeneous right nostril opacity. CECT: anterior right nasal cavity mass with enhancement; no posterior/choanal/nasopharyngeal extension	Intranasal excision (Luc’s forceps); heavy bleeding; attachment cauterized; endoscopy: no posterior extension	NR (6 mo FU)
Szymanska et al. [15] (2006)	57/F	Recurrent right-sided epistaxis and right nasal obstruction, 3 mo	CT: avidly enhancing right nasal cavity/nasopharyngeal mass; bony remodeling/erosion. MRI: avid enhancement, flow-voids. Angio: internal maxillary feeder	Preoperative selective embolization of internal maxillary artery with PVA; en bloc removal via sublabial degloving approach	NR (6 y FU)
Sarafoleanu et al. [16] (2011)	56/M	Progressive persistent nasal obstruction (12 mo) + intermittent mucous rhinorrhea + headache; no epistaxis	CECT: intensely enhancing mass (nasopharynx + both nasal cavities) to soft-palate margin; posterior septal osteolysis; no sphenoid/PPF/ITF extension. Angio: hypervascular; anterior internal maxillary feeder; embolization not feasible	External carotid artery ligation (pre-op) + lateronasal rhinotomy; complete en bloc excision	NR (3 y FU)
Zhang et al. [17] (2015)	72/M	Continuous headaches, right nasal epistaxis, right nasal obstruction and a decreased sense of smell for 3 mo	CT/MRI: strongly enhancing mass with extension to right maxillary sinus, ethmoid sinuses, parapharyngeal space and infratemporal fossa	Endoscopy-assisted sublabial and buccolabial approach with preoperative embolization and incomplete resection followed by subsequent resection	NR (6 mo FU)
Delides et al. [18] (2017)	31/M	Nasal obstruction (1 mo) + epistaxis (preceding 2 weeks)	CT/MRI: right nasal cavity mass protruding to nasopharynx; Proteus-related distorted anatomy	Endoscopic removal under GA (cold instruments); attachment at posterior end of middle turbinate, transected and coagulated; no preoperative angiography/embolization performed	SF (2 y FU)
Raza et al. [19] (2017)	50/M	Right-sided nasal obstruction (2.5 mo) + 2 episodes of epistaxis + diplopia (2 mo) + right frontal/periorbital pain (15 days)	CT (non-contrast): Polypoid mass in right posterior nasal cavity with medial maxillary sinus bulge, Eustachian tube obliteration (R > L). CEMRI: enhancing lobulated mass involving sphenoid sinus/nasopharynx/nasal cavity/posterior ethmoid; extension to clivus/pituitary fossa/pterygoid compartment; minor orbital apex and ACF floor extension	Wilson’s incision; palatal mucosa and periosteum elevated; mass excised (authors describe intracranial extension and removal of intracranial component); palate sutured. No angiography/embolization reported	FU not reported
McGarey et al. [20] (2018)	32/M	Severe nasal hemorrhage after biopsy of a nasopharyngeal mass	CT: posterior nasal cavity/nasopharyngeal mass with pterygopalatine fossa widening	Endoscopic medial maxillectomy + left internal maxillary artery ligation, enabling near-total resection	At 6-mo FU, granulation polyps on biopsy
Ralli et al. [21] (2018)	68/F	18-mo nasal obstruction + episodes of left-sided hearing loss + mucus discharge in nasopharynx + occasional headaches + snoring	CEMRI: polypoid 3.2 × 2.6 cm lesion in posterior nasal cavity extending to nasopharynx; no invasion of nasopharyngeal roof/posterior wall; no upper-neck lymphadenopathy	Functional transnasal endoscopic removal under GA; intraoperative bleeding described as “consistent”	NR (3 y FU)
Stubbs et al. [22] (2019)	62/M	2 y history of decreased sense of smell, increasing right-sided nasal congestion, and recurrent right-sided epistaxis requiring ED control	CT: avidly enhancing right nasal cavity mass with maxillary sinus/masticator space/nasopharynx/orbit extension. MRI: perineural intracranial extension to middle cranial fossa/cavernous sinus	Bilateral medial maxillectomy, sphenoethmoidectomy, frontal sinusotomy, right middle turbinectomy, and posterior septectomy	Residual tumor at 3 mo FU
Rahmadiyanto et al. [23] (2022)	62/M	Bilateral nasal obstruction + anosmia + nasal discharge + epistaxis + nasal voice (12 mo); hearing decrease, blurred vision; dysphagia (1 mo)	CTA: massive destructive sinonasal mass (both nasal cavities + all sinuses) with skull base/orbital extension and frontal subdural involvement. Angio: bilateral IMA + ophthalmic feeders.	Pre-op embolization of bilateral internal maxillary arteries; multidisciplinary joint surgery: medial maxillectomy + extended Killian right lateral rhinotomy + neurosurgical resection/duroplasty (fascia lata) + orbital component excision + reconstruction (polypropylene mesh)	Residual tumor + intracranial abscess susp. on FU; patient asymptomatic
Choi et al. [24] (2023)	32/M	Recurrent bilateral epistaxis + progressive nasal obstruction over 2 y; severe episodes required bilateral nasal packing; severe headaches with photophobia/phonophobia	CT: opacification in left posterior nasal cavity/nasopharynx with vidian canal involvement/expansion. MRI: 1.6 × 2.5 × 3.1 cm T2 hyperintense, T1 hypointense, avidly enhancing lesion with flow voids	Endoscopic resection without preoperative angiography/embolization; tumor extended from left sphenoid face into vidian canal and pterygoid fossa; left internal maxillary artery identified and ligated; en bloc resection with negative margins	NR on FU endoscopy
Kaur et al. [25] (2023)	35/M	Recurrent epistaxis (2 y) + mucopurulent discharge (3 mo); severe episodes (packing/transfusion); headache + visual field reduction; acromegalic features	CT PNS: remodeling/destruction suggestive of angiofibroma (reported grade 2A). MRI brain: pituitary macroadenoma ~4 × 3.7 × 3 cm, chiasmal compression; SWI blooming.	Two-stage endoscopic management: (1) FESS with excision of a vascular mass arising from the right middle turbinate; (2) ~6 weeks later, endoscopic transnasal transsphenoidal excision of pituitary macroadenoma	NR in regular FU
Gozgec et al. [26] (2025)	77/M	Left-sided nasal obstruction, dyspnea, and intermittent epistaxis; exam showed a mass filling the left nasal cavity extending into the nasopharynx	CECT: ~7 × 6 × 4 cm enhancing left nasal cavity/nasopharyngeal mass with septal + medial maxillary wall destruction. CEMRI: T1 low/T2 heterogeneous with flow-voids; origin left lateral nasopharyngeal wall; ethmoid extension	Not performed (patient declined operation)	Radiotherapy
This report (2026)	34/M	Tracheostomy dysfunction with acute respiratory insufficiency; incidental nasopharyngeal mass; post-op hypoparathyroidism with chronic hypocalcemia and Fahr-like calcifications	MRI: 37 × 33 × 32 mm hypervascular mass with bone remodeling; right PPF/sphenopalatine region. DSA: right sphenopalatine feeders	Endovascular embolization + endoscopic resection	NR on FU imaging

NR = no recurrence; FU = follow-up; mo = months; y = years; CECT = contrast-enhanced computed tomography; CEMRI = contrast-enhanced magnetic resonance imaging; CT PNS = computed tomography of the paranasal sinuses; CTA = computed tomography angiography; DSA = digital subtraction angiography; PVA = polyvinyl alcohol; PPF = pterygopalatine fossa; ITF = infratemporal fossa; IMA = internal maxillary artery; GA = general anesthesia; ED = emergency department; ACF = anterior cranial fossa; SWI = susceptibility-weighted imaging.

## Data Availability

The original contributions presented in the study are included in the article, further inquiries can be directed to the corresponding author.

## References

[1] Hodges J.M., McDevitt A.S., Ali A.I.E.S., Sebelik M.E. (2010). Juvenile Nasopharyngeal Angiofibroma: Current Treatment Modalities and Future Considerations. Indian J. Otolaryngol. Head. Neck Surg..

[2] Garofalo P., Pia F., Policarpo M., Tunesi S., Valletti P.A. (2015). Juvenile Nasopharyngeal Angiofibroma: Comparison between Endoscopic and Open Operative Approaches. J. Craniofac. Surg..

[3] Grezenko H., Sobhan A.M., Waqas M., Papuashvili P., Patel V.K., Khan R. (2024). Juvenile Nasopharyngeal Angiofibroma: A Case Study on the Diagnostic and Surgical Challenges in an Adolescent Male. Cureus.

[4] Nonogaki S., Campos H.G.A., Butugan O., Soares F.A., Mangone F.R.R., Torloni H., Mitzi Brentani M. (2010). Markers of Vascular Differentiation, Proliferation and Tissue Remodeling in Juvenile Nasopharyngeal Angiofibromas. Exp. Ther. Med..

[5] Huang Y., Liu Z., Wang J., Sun X., Yang L., Wang D. (2014). Surgical Management of Juvenile Nasopharyngeal Angiofibroma: Analysis of 162 Cases from 1995 to 2012. Laryngoscope.

[6] de Arruda A.C.G., Guerra A.C.D.Z., Pessoa C.H., Marquezine G.F., Delfino V.D.A. (2021). Hypoparathyroidism and Fahr’s Syndrome: Case Series. J. Bras. Nefrol..

[7] Monfrini E., Arienti F., Rinchetti P., Lotti F., Riboldi G.M., Monfrini E., Arienti F., Rinchetti P., Lotti F., Riboldi G.M. (2023). Brain Calcifications: Genetic, Molecular, and Clinical Aspects. Int. J. Mol. Sci..

[8] Converse A., Thomas P. (2021). Androgens Promote Vascular Endothelial Cell Proliferation through Activation of a ZIP9-Dependent Inhibitory G Protein/PI3K-Akt/Erk/Cyclin D1 Pathway. Mol. Cell. Endocrinol..

[9] Chistiakov D.A., Myasoedova V.A., Melnichenko A.A., Grechko A.V., Orekhov A.N. (2018). Role of Androgens in Cardiovascular Pathology. Vasc. Health Risk Manag..

[10] Cleere E.F., McLoughlin L., Lacy P.D. (2023). ‘Juvenile’ Nasal Angiofibroma Presenting in Adulthood. BMJ Case Rep..

[11] Midilli R., Karci B., Akyildiz S. (2009). Juvenile Nasopharyngeal Angiofibroma: Analysis of 42 Cases and Important Aspects of Endoscopic Approach. Int. J. Pediatr. Otorhinolaryngol..

[12] Acharya S., Naik C., Panditray S., Dany S.S. (2017). Juvenile Nasopharyngeal Angiofibroma: A Case Report. J. Clin. Diagn. Res..

[13] Taylor K., DiBlasi M., Pedersen E., Shahsavari N. (2024). The Radiological Perspective of Juvenile Nasopharyngeal Angiofibroma: A Case Report. Radiol. Case Rep..

[14] Shah N., Hathiram B.T., Dwivedi A., Sheth K., Grewal D.S., Behl N.K. (2000). Nasopharyngeal Angiofibroma-an Unusual Origin & Presentation. Indian J. Otolaryngol. Head. Neck Surg..

[15] Szymańska A., Korobowicz E., Goła̧bek W. (2006). A Rare Case of Nasopharyngeal Angiofibroma in an Elderly Female. Eur. Arch. Oto-Rhino-Laryngol. Head. Neck.

[16] Sarafoleanu C., Enache R., Rhinol I.S.-R.J. (2011). Undefined Unusual Case of Nasopharyngeal Angiofibroma in Adult Male Patient. Rom. J. Rhinol..

[17] Zhang H.K., Wang J.J., Liu Z.F., Wang D.H. (2015). Management of Nasopharyngeal Angiofibroma in a 72-Year-Old Male through a Sublabial and Buccolabial Incision Approach: A Case Report and Literature Review. Oncol. Lett..

[18] Delides A., Panayiotides J.G., Kaberos A., Giotakis I. (2017). Nasopharyngeal Angiofibroma in an Adult with Proteus Syndrome. First Reported Case. Hippokratia.

[19] Raza S.S., Owais S.M., Zahid S., Haq I.U., Manan H., Qasim S.F., Ullah F., Hijazi A.S., Khan O.R. (2017). Angiofibroma in A 50-Year-Old Patient. J. Ayub Med. Coll. Abbottabad.

[20] McGarey P.O., David A.P., Payne S.C. (2018). Nasopharyngeal Angiofibroma in a 32-Year-Old Man. Case Rep..

[21] Ralli M., Fusconi M., Visconti I.C., Martellucci S., de Vincentiis M., Greco A. (2018). Nasopharyngeal Angiofibroma in an Elderly Female Patient: A Rare Case Report. Mol. Clin. Oncol..

[22] Stubbs V.C., Miller L.E., Parasher A.K., Glicksman J.T., Adappa N.D., Palmer J. (2019). Nasopharyngeal Angiofibroma: A Forgotten Entity in Older Patients. Clin. Med. Insights Case Rep..

[23] Rahmadiyanto Y., Romdhoni A.C. (2022). Multidiscipline Management of Giant Reccurent Nasopharyngeal Angiofibroma Which Extends to Paranasal Sinuses, Orbita, and Intracranial in Adult. Int. J. Surg. Case Rep..

[24] Choi S., Zhang V.J., Zhu X., Ito C.J. (2023). Nasopharyngeal Angiofibroma in an Adult Male: A Case Report and Review of the Literature. Cureus.

[25] Kaur J., Deshmukh P.T., Jain S., Singh C.V., Gaurkar S.S. (2023). A Rare Association of Pituitary Macroadenoma with Nasopharyngeal Angiofibroma: A Case Report. Cureus.

[26] Gozgec E., Ogul H., Aktan B. (2025). Unexpected Tumor in an Elderly Patient: Nasopharyngeal Angiofibroma. Ear Nose Throat J..

[27] Janakiram T.N., Sharma S.B., Samavedam U.C., Deshmukh O., Rajalingam B. (2017). Imaging in Juvenile Nasopharyngeal Angiofibroma: Clinical Significance of Ramharan and Chopstick Sign. Indian J. Otolaryngol. Head. Neck Surg..

[28] Alimli A.G., Ucar M., Oztunali C., Akkan K., Boyunaga O., Damar C., Derinkuyu B., Tokgöz N. (2016). Juvenile Nasopharyngeal Angiofibroma: Magnetic Resonance Imaging Findings. J. Belg. Soc. Radiol..

[29] Vlăescu A.N., Ioniţă E., Ciolofan M.S., Mogoantă C.A., Voiosu C., Rusescu A., Hainăroşie R. (2022). Current Approach of Juvenile Nasopharyngeal Angiofibroma: A Case Series. Rom. J. Morphol. Embryol..

[30] Orloff L.A., Wiseman S.M., Bernet V.J., Fahey T.J., Shaha A.R., Shindo M.L., Snyder S.K., Stack B.C., Sunwoo J.B., Wang M.B. (2018). American Thyroid Association Statement on Postoperative Hypoparathyroidism: Diagnosis, Prevention, and Management in Adults. Thyroid.

[31] Clarke B.L. (2022). Hypoparathyroidism: Update of Guidelines from the 2022 International Task Force. Arch. Endocrinol. Metab..

[32] Bollerslev J., Buch O., Cardoso L.M., Gittoes N., Houillier P., Van Hulsteijn L., Makay O., Marcocci C., Pallais J.C., Pilz S. (2025). Revised European Society of Endocrinology Clinical Practice Guideline: Treatment of Chronic Hypoparathyroidism in Adults. Eur. J. Endocrinol..

[33] Kalampokini S., Georgouli D., Dadouli K., Ntellas P., Ralli S., Valotassiou V., Georgoulias P., Hadjigeorgiou G.M., Dardiotis E., Xiromerisiou G. (2021). Fahr’s Syndrome Due to Hypoparathyroidism Revisited: A Case of Parkinsonism and a Review of All Published Cases. Clin. Neurol. Neurosurg..

[34] Berrabeh S., Messaoudi N., Elmehraoui O., Assarrar I., Karabila I., Jamal A., Zeryouh N., Rouf S., Latrech H. (2023). Hypoparathyroidism and Fahr’s Syndrome: A Case Series. Cureus.

